# Investigation of the protective effect of visnagin against cadmium toxicity in rat nephropathy

**DOI:** 10.1007/s00210-026-05440-4

**Published:** 2026-05-14

**Authors:** Selim Demirtas, Gul Sahika Gokdemir, Ugur Seker

**Affiliations:** 1 Faculty of Medicine, Department of Histology and Embryology, Mardin Artuklu University, Mardin, Türkiye; 2 Faculty of Medicine, Department of Physiology, Mardin Artuklu University, Mardin, Türkiye

**Keywords:** Visnagin, Cadmium, Nephrotoxicity, Oxidative Stress, Inflammation, Rats

## Abstract

Cadmium (Cd) is a widespread environmental pollutant that accumulates primarily in the kidneys and induces nephrotoxicity through oxidative stress, inflammation, and structural damage. Visnagin, has been reported to exhibit antioxidant and anti-inflammatory properties. This study aimed to investigate the protective effects of visnagin against cadmium-induced renal toxicity in rats. Twenty-eight adult Wistar albino rats were randomly assigned into four groups (*n* = 7): Control, Cadmium (Cd; 3 mg/kg CdCl₂, i.p.), Visnagin + Cadmium (Vis + Cd; 60 mg/kg visnagin orally + CdCl₂), and Visnagin (Vis;60 mg/kg visnagin orally). Treatments were administered daily for 15 days. Renal tissues were collected for histopathological examination using H&E and PAS staining. To determine anti-Bax and anti-Bcl-2 immunreactivity were applied immunohistochemistry. Oxidative stress parameters (MDA, SOD, GPX1) and inflammatory cytokines (IL-1β, IL-6) were analyzed in kidney tissue by ELISA. Serum BUN and creatinine levels were measured to evaluate renal function. Data were statistically analyzed using appropriate parametric and nonparametric tests. Cadmium exposure caused significant renal damage, characterized by tubular dilation, glomerular atrophy, basement membrane thickening, increased inflammatory infiltration, elevated MDA, IL-1β, and IL-6 levels, reduced SOD and GPX1 activities, and increased serum BUN and creatinine levels (*p* < 0.05). Visnagin treatment significantly attenuated Cd-induced histopathological alterations, suppressed oxidative stress and inflammatory responses, and improved renal function parameters. Visnagin reduced apoptotic damage by reversing CD-induced Bax increase and Bcl-2 loss. In conclusion, visnagin exerts a significant nephroprotective effect against cadmium-induced kidney injury through antioxidant, anti-inflammatory, and functional preservation mechanisms.

**Graphical Abstract:**

**Figure:**
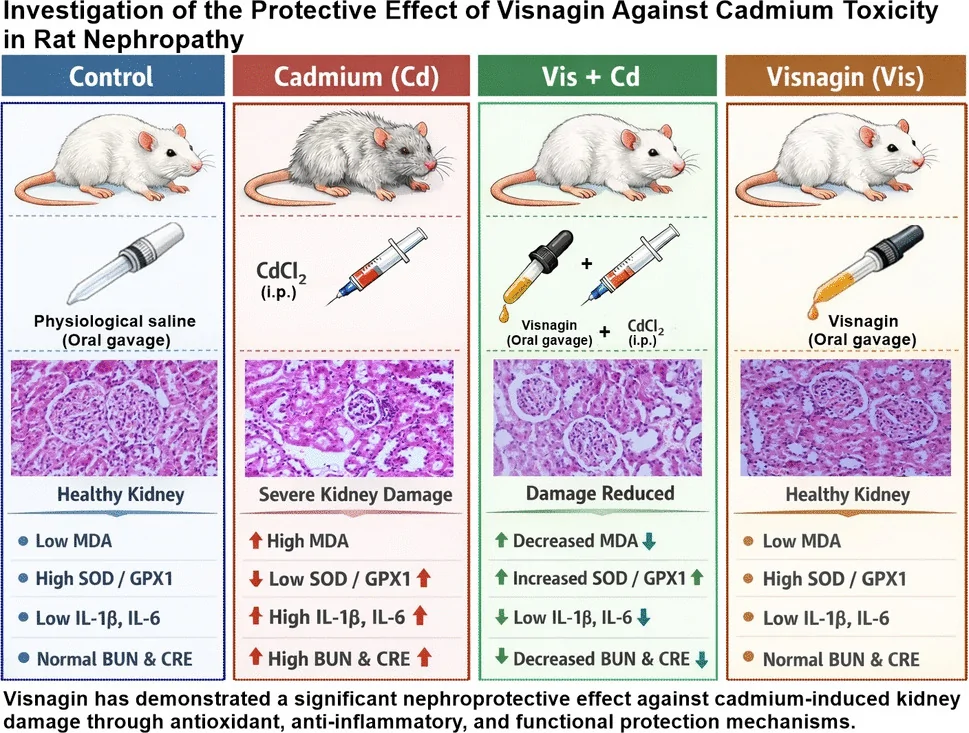

## Introduction

Cadmium, found in very limited amounts in nature, has become a significant pollutant, particularly in the last century, due to industrial production (Huff et al. 2007; Charkiewicz et al. 2023) It is used directly in nickel–cadmium batteries, metal plating, the paint industry, PVC stabilizers, alloys, and the electronics sector; it also appears as an undesirable impurity in phosphate fertilizers, detergents, and refined petroleum products (Coşan et al. 2017).

Cadmium exposure primarily occurs through the respiratory and digestive systems, and it has been reported to cause irritation to the lungs, liver, and kidneys (Wan et al. 2022). Furthermore, its soluble form, CdCl₂, causes toxicity in lungs, liver, and kidneys, as well as the brain, testes, and heart (Rinaldi et al. 2017). Its introduction into the food chain through agricultural products, drinking water pollution, and smoking also significantly increase cadmium intake (Aqeel et al. 2020; Wróblewski et al. 2024).

Cadmium is transported in the body by complexing with metallothionein, and after its breakdown in the proximal tubules, free cadmium ions are released. These ions cause cell damage through oxidative stress, inflammation, and apoptosis (Järup 2002; Sanjeev et al. 2019; Yan and Allen 2021). Studies on this topic indicate that the primary mechanisms of Cd toxicity are increased reactive oxygen species (ROS) production and weakened antioxidant defense systems (Hernández-Cruz et al. 2022; Ijaz et al. 2023).

Cadmium exposure leads to severe oxidative stress in renal tissues by significantly increasing ROS and malondialdehyde (MDA) levels. This toxic metal weakens cellular defense mechanisms by suppressing the activities of key antioxidant enzymes such as superoxide dismutase (SOD), catalase (CAT), and glutathione peroxidase (GPx), as well as related gene expression (Hamza et al. 2025; Ijaz et al. 2025). Cadmium-induced nephrotoxicity results in elevated levels of urea, creatinine, NGAL, and KIM-1, and significant decreases in creatinine clearance and albumin levels, all indicators of renal function. Histopathological findings confirm severe structural damage, including proximal tubule dilation, Bowman's capsule damage, glomerular atrophy, and tubular necrosis. Furthermore, these damages are accompanied by an increase in inflammatory cytokines (NF-κB, TNF-α, IL-6) and the triggering of apoptosis, thus disrupting the integrity of kidney tissue (Hamza et al. 2025; Ijaz et al. 2025).

In recent years, the protective effects of natural compounds against heavy metal toxicity have attracted interest. Visnagin is a prominent natural molecule in this field. Its antioxidant and anti-inflammatory properties have been reported to protect kidney epithelial cells, exhibit diuretic effects and have a positive impact on the cardiovascular system (Wan et al. 2023). It may also be a suitable treatment for heavy metal-caused toxicities, such as Cd (Ajarem et al. 2021).

Despite these documented benefits, the specific role of visnagin in mitigating Cd-induced renal structural damage and its influence on the underlying apoptotic pathways have not yet been fully elucidated. While previous studies have addressed visnagin’s general nephroprotective potential, its targeted efficacy in preventing Cd-driven basement membrane thickening and tubular atrophy remains a critical knowledge gap. This study is novel as it provides a comprehensive investigation into how visnagin regulates the Bax/Bcl-2 mediated apoptotic signaling alongside a detailed histopathological and biochemical evaluation in a rat model of Cd toxicity. Therefore, this study aimed to investigate the toxic effects of cadmium exposure on rat kidney tissue and the potential protective role of visnagin, thereby offering a more nuanced understanding of its therapeutic mechanisms.

## Methods

This study was submitted to the Dicle University Health Sciences Application and Research Center Animal Experiments Local Ethics Committee (DÜHADEK) and received ethical approval with the date and decision number 26/06/2025—10.

### Animals and experimental design

A total of 28 healthy adult Wistar Albino rats were randomly assigned to four groups of 7 animals each. The animals were housed at 22 ± 2 °C, under a 12 h light/12 h dark cycle and provided standard pellet chow and tap water ad libitum. Groups: Group I (Control)-fed a standard diet and tap water and received 0.5 ml of physiological saline via oral gavage; Group II (Cd)-received 3 mg/kg cadmium chloride (202,908, CAS No: 10108—64—2, purity: 99.99%, Merck Millipore) solution daily, administered intraperitoneally (i.p.); Group III (Vis + Cd)-received 60 mg/kg Visnagin (34,140, CAS No: 82—57—5, purity: 98%, Cayman Chem) dissolved in 0.5 ml of isotonic solution orally together with 3 mg/kg CdCl₂ (i.p.) daily; Group IV (Vis)-administered 60 mg/kg Visnagin dissolved in 0.5 ml of isotonic solution orally. All experimental treatments were repeated daily for 15 days. The duration of the study was selected in accordance with previously reported cadmium-induced renal injury protocols (Sanjeev et al. 2019). On day 16 of the experiment, rats were anesthetized with a combination of 9 mg/kg xylazine and 90 mg/kg ketamine HCl after a 12-h fast. After the animals were sacrificed, blood and kidney tissue samples were collected. A portion of the kidney tissue was fixed in 10% formalin and embedded in paraffin blocks following routine histological procedures. The remaining kidney tissue was frozen in liquid nitrogen and stored at −80 °C for subsequent analyses.

### Histological examination

5 µm-thick sections taken from paraffin blocks were stained with Hematoxylin–Eosin (H&E) and Periodic Acid-Schiff (PAS). Sections were examined under a light microscope (Carl Zeiss Axiolab 5, Carl Zeiss, Oberkochen, Germany), and histological structures and glomerular morphology of renal tissue were evaluated using calibrated image analysis software (Zeiss Zen 3.7, Carl Zeiss, Oberkochen, Germany). Ten slides were prepared from each kidney. Only the cortical regions of the kidneys were examined and glomerular changes, hemorrhage and inflammatory parameters were evaluated by adapting the same 0—5 percentile scale to measure the extensive effects of Cd toxicity following the method described in the relevant literature (Kuruş et al. 2005). The severity of lesions was scored on a scale of 0–5, corresponding to the approximate percentage of tissue affected (0 = no injury; 1 = ≤ 10%; 2 = 10—25% of tubules injured; 3 = 26—50% of tubules injured; 4 = 51—75% of tubules injured; 5 = > 75% of tubules injured) (Kuruş et al. 2005). All evaluations were performed in a blinded manner under light microscope (Carl Zeiss Axiolab 5, Carl Zeiss, Oberkochen, Germany).

### Immunohistochemical examination

To determine apoptotic activity, paraffin sections with a thickness of 5 µm were deparaffinized in xylene, rehydrated by passing through graduated alcohol series, and then heated in citrate buffer (pH 6.0) in a microwave oven for 20 min for antigen retrieval. Endogenous peroxidase activity was blocked with 3% H₂O₂ for 10 min, and the sections were treated with Ultra V Block to prevent non-specific binding. Primary antibodies, anti-Bax Ab (AF0120, Affinity Biosciences) and anti-Bcl-2 Ab (AF6139, Affinity Biosciences), were incubated at 37 °C for 1 h at a 1/100 dilution. HRP-conjugated secondary antibodies were applied, the immune reaction was visualized with 3,3'-diaminobenzidine (DAB) chromogen, and counterstained with hematoxylin. The evaluation was based on relevant literature (Pila et al. 2023). Staining intensity was rated on a scale of 0 (none), 0.5 (trace), 1 (weak), and 2 (strong) for each animal, using 10 randomly selected microscopic structures. All evaluations were performed in a blinded manner under light microscope (Carl Zeiss Axiolab 5, Carl Zeiss, Oberkochen, Germany). The score was analyzed semi-quantitatively using the formula:

$$Score: \frac{\left(\mathrm{0,5}x{N}_{\mathrm{0,5}}+1 x {N}_{1}+2x{N}_{2}\right)}{N}$$

### Biochemical and molecular analyses

Biochemical analyses were performed to evaluate oxidative stress parameters in kidney tissue. All analyses were performed using commercially available enzyme-linked immunosorbent assay (ELISA) kits, and samples were prepared according to the manufacturer's instructions. For ELISA analyses, homogenates were prepared from kidney tissues stored at −80 °C. Kidney tissues were removed from the freezer, placed in a glass tube containing PBS at a 1:10 (w/v, pH: 7.2) ratio, and homogenized on ice at 30,000 g for 60 s using a tissue homogenizer (Bandelin, UW 2070, Sigma, St. Louis, MO). Homogenates were placed in centrifuge tubes and centrifuged at 4,200 g for 10 min at 4 °C. The supernatants were transferred to an Eppendorf tube and the precipitates were discarded. Interleukin-1 beta (IL-1β) levels (Sun Red, Shanghai Sunred Biological Technology, China; Catalog Number: 201—11—0120), Interleukin-6 (IL-6) levels (Sun Red, Shanghai Sunred Biological Technology, China; Catalog Number: 201—11—0136), GPx1 enzyme activities (ATLAS Biotechnology Inc., Turkey; Catalog Number: ABT2557Ra), SOD levels (Shanghai YL Biotechnology Inc., China; Catalog Number: YLA0115RA), and MDA levels (ATLAS Biotechnology Inc., Turkey; Catalog Number: ABT10165Ra) were measured by ELISA in kidney tissue supernatants using specific ELISA kits according to the manufacturer's instructions. The supernatant was read spectrophotometrically at 450 nm using a microplate reader (Biochrom, Anthos Zenyth 200). Results were given as ng/mL or pg/mL.

Blood samples from each animal were centrifuged at 1500 g for 10 min to separate the serum (Megafuge STPlus, Thermo Scientific, Waltham, MA). Serum supernatants were collected into sterile tubes, and Blood Urea Nitrogen (BUN) and creatinine (CRE) levels were analyzed on a biochemical autoanalyzer (Architect c8000; Abbott, Wiesbaden, Germany).

### Statistical analysis

The sample size was determined using power analysis. Assuming a large effect size Cohen’s d = 1.2), α = 0.05, and power (1 − β) = 0.80, a minimum of 6 animals per group was calculated as sufficient. To account for potential experimental losses, 7 animals per group were included.

Statistical analyses were performed using IBM SPSS Statistics version 25.0. Data distribution was assessed using the Shapiro–Wilk and Kolmogorov–Smirnov tests. For intergroup comparisons, One-Way Analysis of Variance (ANOVA) followed by Tukey’s post-hoc test was applied to variables showing a normal distribution. For data that did not conform to a normal distribution, the Kruskal–Wallis test was used. Results are presented as mean ± standard error of the mean (SEM), and a p-value < 0.05 was considered statistically significant.

## Results

### Histopathology

Histopathological examination of kidney tissue revealed a significant improvement in kidney damage parameters in the Cd-induced group compared to the control group. Tubular damage, glomerular changes, hemorrhage, and inflammatory cell infiltration scores were significantly higher (*p* < 0.05). In contrast, the Cd + Vis group showed a significant reduction in Cd-induced kidney damage. All evaluated histopathological parameters showed significant improvement in the Vis + Cd group compared to the Cd group, with a reduction of approximately 35% to 50% in damage scores (*p* < 0.05). No statistically significant difference was observed between the Vis group and the control group for any histopathological parameter (*p* > 0.05). Comparative histopathological findings are summarized in Table 1.

**Table 1:** Comparative histopathological evaluation of kidney injury parameters across experimental groups

Parameter	Control	Cd	Vis + Cd	Vis
Tubular injury	0.0 ± 0.0	2.3 ± 0.7^a^	1.1 ± 0.6^b^	0.1 ± 0.3^c^
Glomerular alterations	0.0 ± 0.0	1.4 ± 0.5^a^	0.6 ± 0.2^b^	0.0 ± 0.0
Hemorrhage	0.0 ± 0.0	1.2 ± 0.4^a^	0.5 ± 0.1^b^	0.0 ± 0.0
Inflammatory infiltration	0.0 ± 0.0	2.2 ± 0.7^a^	1.0 ± 0.6^b^	0.0 ± 0.0

Data are presented as mean ± SD. Statistical analysis was performed using one-way ANOVA followed by Tukey post-hoc test

^a^Indicates a significant difference compared with the Control group (*p* < 0.05)

^b^Indicates a significant difference compared with the Cd group (*p* < 0.05). ^c^There is no significant difference when compared to the control group (*p* = 0.331). *Cd* Cadmium chloride, *Vis + Cd* Visnagin + Cadmium chloride, *Vis* Visnagin

**Fig. 1:**
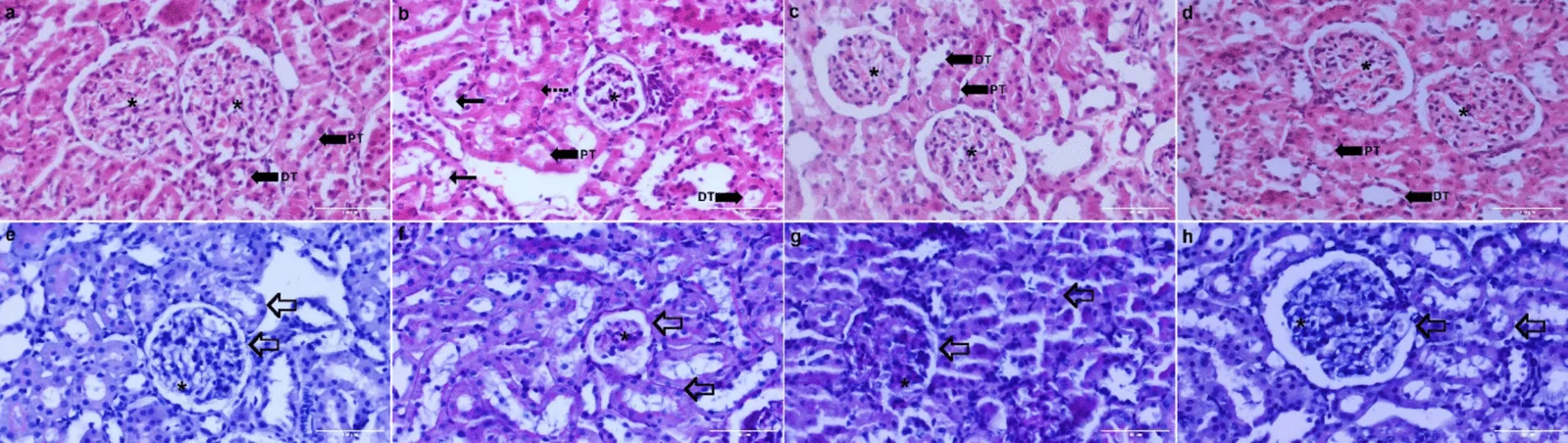
Histopathological evaluation of kidney tissues by hematoxylin–eosin (a–d) and periodic acid-Schiff (**e–h**) staining. (**a**,** e**) Control group shows normal glomerular architecture and preserved tubular structures with no pathological changes. (**b**,** f**) Cd group demonstrates: (**i**) H&E—marked tubular dilation (arrows), epithelial degeneration with increased eosinophilia (segmented arrow), and glomerular atrophy (asterisks); (ii) PAS—basement membrane thickening (hollow arrows) and glomerular structural disruption (asterisks). (**c**,** g**) Cd + Vis group shows intermediate pathology between Cd and control groups. Glomerulus (asterisks). (**d**,** h**) Vis group appears normal, similar to controls. DT: Distal tubule, PT: Piroximal tubule, basement membranes (hollow arrows). Cd: Cadmium chloride; Vis + Cd: Visnagin + Cadmium chloride; Vis: Visnagin. Magnification × 400

H&E and PAS staining performed under the microscope also clearly revealed these differences (Fig. 1). While the kidney tissue in the control group maintained its normal structure (Fig. 1a-e), in the Cd group, marked tubular dilation, glomerular atrophy, epithelial cell deterioration, and an increase in tissue eosinophilia were notable (Fig. 1b). These pathological findings were significantly reduced in the visnagin-treated group (Fig. 1c). In the visnagin-only group, the tissue structure appeared healthy, similar to the control group (Fig. 1d-h).

PAS staining results also confirmed the histopathological alterations in the Cd group: basement membrane thickening and glomerular structural deterioration were clearly visible (Fig. 1f). In the visnagin-treated rats, these adverse changes were significantly reduced, and the kidney tissue was preserved (Fig. 1g).

### Immunohistochemistry

**Fig. 2:**
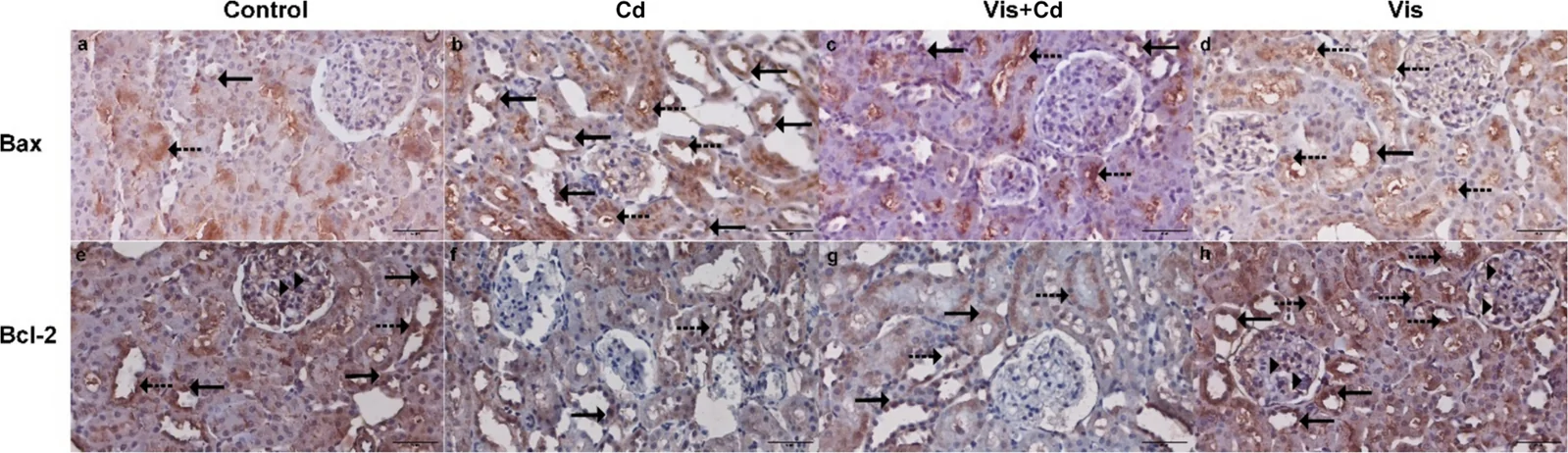
Immunohistochemical staining images of Bax and Bcl-2 in kidney tissue. In the upper panel, Bax expression is observed at basal levels in the control (**a**) and Visnagin (**d**) groups, while a strong positive reaction is seen in the tubular epithelia of the cadmium (**b**) group. In the Vis + Cd (**c**) group, Bax positive staining is observed to be significantly reduced compared to the cadmium group. In the lower panel, Bcl-2 expression shows strong positivity in the tubules and around the glomerulus in the control (**e**) and Visnagin (**h**) groups, while this expression is dramatically suppressed in the cadmium (**f**) group. In the Vis + Cd (**g**) group, Visnagin administration appears to increase Bcl-2 immunoreactivity. Distal tubules (arrows), Piroximal tubules (segmented arrows), Mesangial cells (arrowheads). Cd: Cadmium chloride; Vis + Cd: Visnagin + Cadmium chloride; Vis: Visnagin. Magnification × 400

**Fig. 3:**
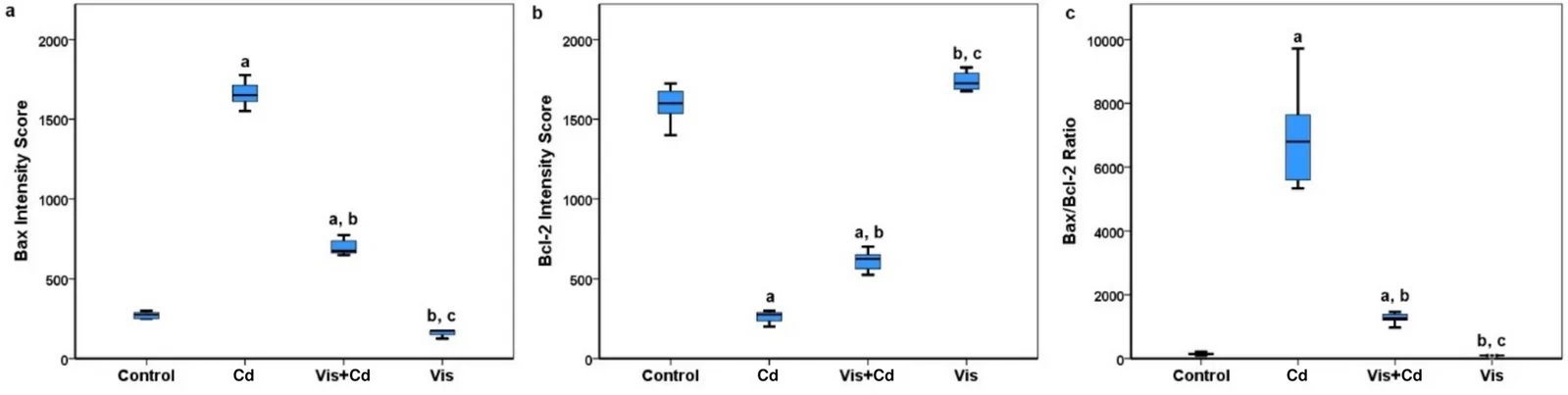
Graphical distribution of (**a**) Bax Intensity Score, (**b**) Bcl-2 Intensity Score, and (**c**) Bax/Bcl-2 Ratio in the groups. Cadmium administration was found to cause a significant increase in Bax and the Bax/Bcl-2 ratio, and a significant decrease in Bcl-2 levels. Visnagin treatment significantly reduced the Bax/Bcl-2 ratio compared to the Cd group (*p* < 0.05), maintaining apoptotic balance. Letter Markings (*p* < 0.05): ᵃSignificant difference compared to the control group. ᵇSignificant difference compared to the Cd group. ᶜSignificant difference compared to the Visnagin + Cd group. Cd: Cadmium chloride; Vis + Cd: Visnagin + Cadmium chloride; Vis: Visnagin

Both Bax and Bcl-2 expression were observed cytoplasmically in the cells. While Bax expression was observed at basal levels in the control and Visnagin groups, severe and widespread immunoreactivity was observed in the tubular epithelia of the Cd group. In the treatment group, Visnagin exhibited a protective effect by significantly reducing Bax expression. Bcl-2 staining showed strong expression in the control and Visnagin groups, while its expression was almost completely eliminated in the Cd group. In the Visnagin + Cd group, Bcl-2 levels were determined to have increased again compared to the Cd group. In Bcl-2 staining, immunoreactivity was observed in mesangial cells only in the control and visnagin groups. (Figs. 2, 3 and Table 2).

**Table 2:** Statistical comparison of renal tissue Bax, Bcl-2 ıntensity scores and Bax/Bcl-2 Ratios in groups

Parameter	Control	Cd	Vis + Cd	Vis
Bax Intensity score	0,26 ± 0,06	1,66 ± 0,08^a^	0,70 ± 0,05^a,b^	0,17 ± 0,03^b,c^
Bcl-2 Intensity score	1,59 ± 0,11	0,26 ± 0,04^a^	0,61 ± 0,06^a,b^	1,74 ± 0,06^b,c^
Bax/Bcl-2 Ratio	0,15 ± 0,04	6,90 ± 1,63^a^	1,31 ± 0,25^a,b^	0,10 ± 0,02^b,c^

Data are presented as mean ± SD. Statistical analysis was performed using one-way ANOVA followed by Tukey post-hoc test

ᵃSignificantly different compared to the control group (*p* < 0.05)

ᵇSignificantly different compared to the Cd group (*p* < 0.05)

ᶜSignificantly different compared to the Vis + Cd group (*p* < 0.05). *Cd* Cadmium chloride, *Vis* + *Cd* Visnagin + Cadmium chloride, *Vis* Visnagin

The Bax/Bcl-2 ratio, reflecting the tendency of cells to undergo apoptosis, reached its highest score in the Cd group (6,90 ± 1,63), showing a dramatic increase compared to all other groups. In the Visnagin + Cd group (1,31 ± 0,2), this ratio was significantly lower compared to the Cd group (Table 2, Fig. 3).

### Biochemical analyses of oxidative stress and kidney function parameters

**Fig. 4:**
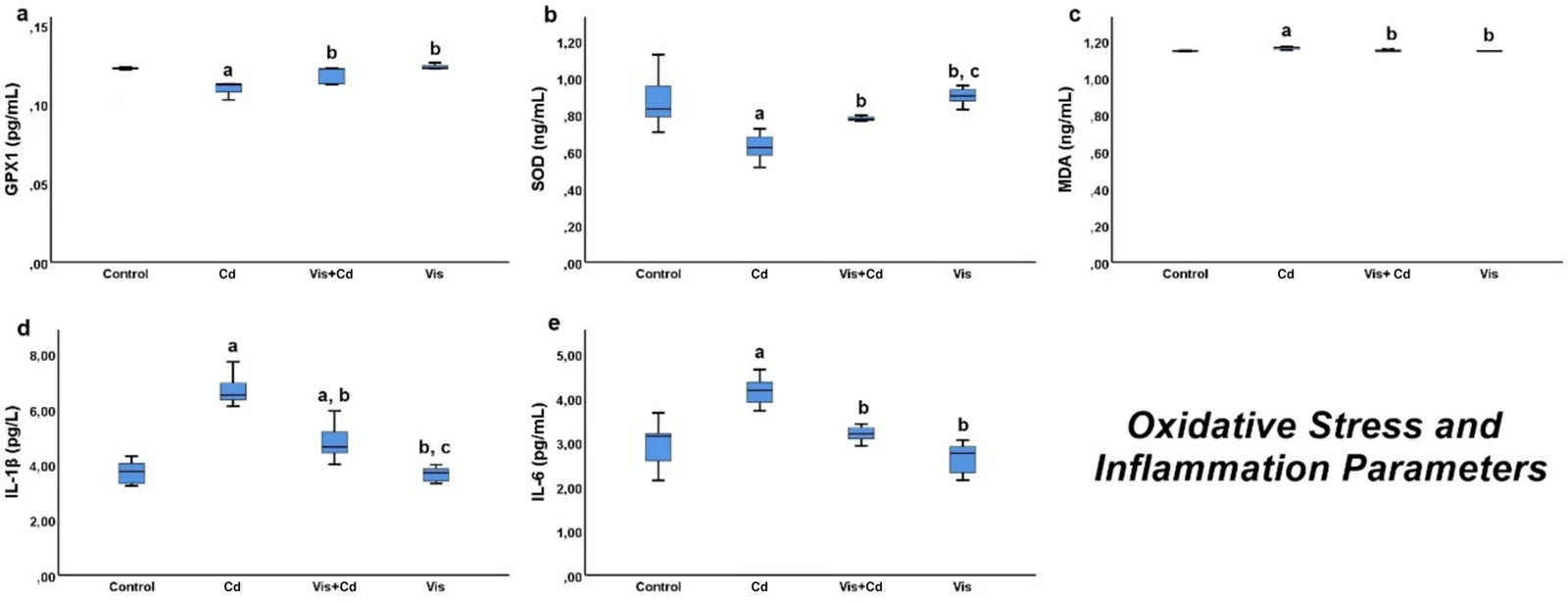
Data are presented as mean ± SD. Statistical analysis was performed using one-way ANOVA followed by Tukey post-hoc test. ^a^Indicates a significant difference compared with the Control group (p < 0.05); ^b^Indicates a significant difference compared with the Cd group (*p* < 0.05). ^a^*p* < 0.05 vs Control; ^b^*p* < 0.05 vs Cd group. ^c^*p* < 0.05 vs Vis + Cd group (**a**) GPX1: Glutathione Peroxidase 1; (**b**) SOD: Superoxide Dismutase; (**c**) MDA: Malondialdehyde; (**d**) IL-1β: Interleukin-1 beta; (**e**) IL-6: Interleukin-6. Cd: Cadmium chloride; Vis + Cd: Visnagin + Cadmium chloride; Vis: Visnagin

**Fig. 5:**
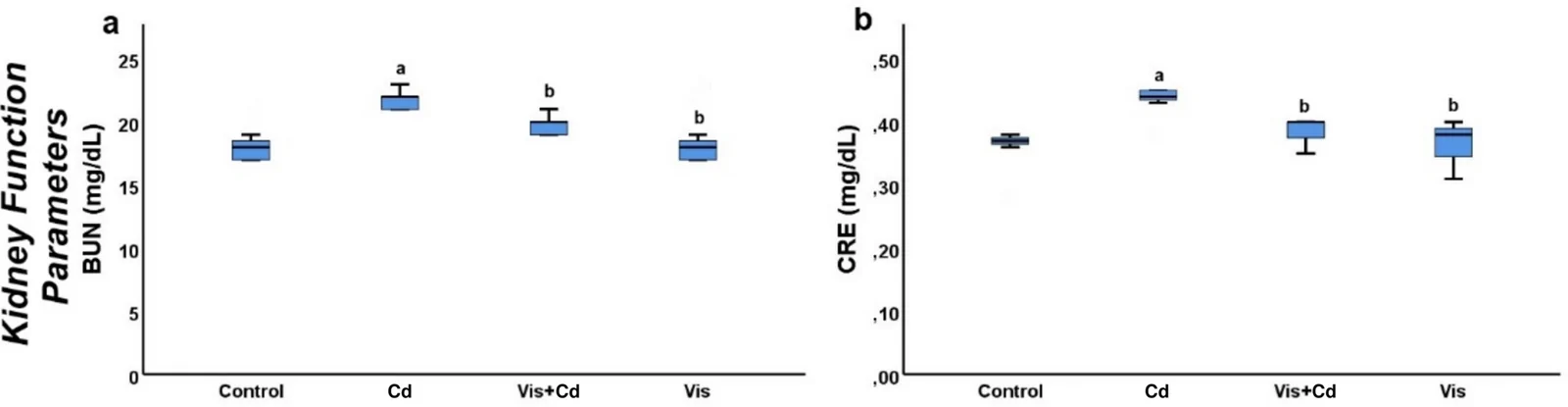
Data are presented as mean ± SD. Statistical analysis was performed using one-way ANOVA followed by Tukey post-hoc test. ^a^Indicates a significant difference compared with the Control group (*p* < 0.05); ^b^Indicates a significant difference compared with the Cd group (*p* < 0.05). ^a^*p* < 0.05 vs Control; ^b^*p* < 0.05 vs Cd group. (**a**) BUN: Blood Urea Nitrogen; (**b**) CRE: Creatinine. Cd: Cadmium chloride; Vis + Cd: Visnagin + Cadmium chloride; Vis: Visnagin

Comparisons of inflammation, oxidative stress and renal function parameters between the experimental groups are presented in Table 3 (Figs. 4, 5).

**Table 3:** Comparative assessment of inflammation, oxidative stress and kidney function parameters between groups

Parameter	Control	Cd	Vis + Cd	Vis
IL-1β (pg/L)	3.71 ± 0.43	6.70 ± 0.55^a^	4.82 ± 0.71^a, b^	3.64 ± 0.27^b, c^
IL-6 (pg/mL)	2.93 ± 0.56	4.15 ± 0.33^a^	3.23 ± 0.26^b^	2.62 ± 0.36^b^
GPX1(pg/mL)	0.12 ± 0.0	0.10 ± 0.0^a^	0.11 ± 0.0^b^	0.12 ± 0.0^b^
SOD(ng/mL)	0.87 ± 0.14	0.62 ± 0.07^a^	0.77 ± 0.03^b^	0.91 ± 0.07^b, c^
MDA(ng/mL)	1.14 ± 0.0	1.16 ± 0.0^a^	0.15 ± 0.0^b^	0.14 ± 0.0^b^
BUN(mg/dL)	18.14 ± 1.46	21.71 ± 0.75^a^	19.71 ± 0.75^b^	18.29 ± 1.79^b^
CRE(mg/dL)	0.35 ± 0.03	0.43 ± 0.03^a^	0.38 ± 0.02^b^	0.36 ± 0.03^b^

Data are presented as mean ± SD. Statistical analysis was performed using one-way ANOVA followed by Tukey post-hoc test.^a^Indicates a significant difference compared with the Control group (*p* < 0.05); ^b^Indicates a significant difference compared with the Cd group (*p* < 0.05). ^a^*p* < 0.05 vs Control; ^b^*p* < 0.05 vs Cd group. ^c^*p* < 0.05 vs Vis + Cd group *GPX1* Glutathione peroxidase 1, *SOD* Superoxide dismutase, *MDA* Malondialdehyde, *BUN* Blood urea nitrogen, *CRE* Creatinine, *Cd* Cadmium chloride, *Vis* + *Cd* Visnagin + Cadmium chloride, *Vis* Visnagin

IL-1β levels in kidney tissues were significantly increased in the Cd group compared to the control group (*p* < 0.001). In contrast, IL-1β levels decreased in the Vis + Cd group but remained higher than in the control group (*p* = 0.003). IL-1β levels in the Vis groups were similar to the Control group (*p* = 0.994). IL-1β levels were significantly decreased in the Cd + Vis and Vis groups compared to the Cd group (*p* < 0.001). IL-1β levels in the Vis group were lower than in the Cd + Vis group (*p* = 0.002).

IL-6 levels in kidney tissues were significantly increased in the Cd group compared to the control group (*p* < 0.001). IL-1β levels in the Vis + Cd and Vis groups were similar to the Control group (*p* = 0.489 and *p* = 491, respectively). A significant decrease in IL-1β levels was observed in the Cd + Vis and Vis groups compared to the Cd group (*p* < 0.001). IL-6 levels were lower in the Vis group compared to the Cd + Vis group (*p* = 0.039). GPX1 levels were significantly decreased in the Cd group compared to the control group (*p* < 0.001). In contrast, GPX1 activity in the Vis + Cd and Vis groups remained similar to the Control group (*p* = 0.137; *p* = 0.971). The Vis + Cd and Vis groups showed a significant increase in GPX1 compared to the Cd group (*p* < 0.001). GPX1 activity was similar between the Vis + Cd and Vis groups (*p* = 0.057).

Similarly, SOD activity was significantly decreased in the Cd group compared to the control group (*p* < 0.001), whereas both the Vis + Cd and Vis groups showed significantly higher SOD levels compared to the Cd group, approaching control values ​​(*p* = 0.176; *p* = 0.863, respectively). The Cd + Vis and Vis groups showed a significant increase in SOD compared to the Cd group (*p* = 0.026; *p* < 0.001, respectively). SOD activity was statistically significantly increased in the Vis group compared to the Cd + Vis group (*p* = 0.037).

In contrast, MDA, an important indicator of lipid peroxidation, was significantly higher in the Cd group compared to the Control group (*p* < 0.001), whereas both the Vis + Cd and Vis groups exhibited significantly lower MDA levels compared to the Cd group, approaching control values ​​(*p* = 0.385; *p* = 1.000, respectively). Both the Vis + Cd and Vis groups had significantly lower MDA levels compared to the Cd group (*p* < 0.001). MDA activity was similar between the Vis + Cd and Vis groups (*p* = 0.446).

Among the kidney parameters, BUN levels were significantly increased in the Cd group compared to the control group (*p* < 0.001). However, both the Vis + Cd and Vis groups approached control values ​​with lower BUN levels compared to the Cd group (*p* = 0.126; *p* = 0.997, respectively). Both the Vis + Cd and Vis groups had significantly lower BUN levels compared to the Cd group (*p* = 0.034; *p* < 0.001, respectively). There was no significant difference in BUN levels between the Vis + Cd and Vis groups (*p* = 0.184) (Fig. 5).

Among the kidney parameters, CRE levels were significantly increased in the Cd group compared to the control group (*p* < 0.001). However, both the Vis + Cd and Vis groups approached control values ​​with lower CRE levels compared to the Cd group (*p* = 0.389; *p* = 0.974, respectively). Both the Vis + Cd and Vis groups had significantly lower CRE levels compared to the Cd group (*p* = 0.021; *p* < 0.001, respectively). There was no significant difference in CRE levels between the Vis + Cdd group and the Vis groups (*p* = 0.639) (Fig. 4).

## Discussion

As the body's primary excretory organ, the kidney is the main target organ for Cd accumulation. Studies have reported that Cd exposure can cause nephrotoxicity, primarily reflected in renal structural destruction and dysfunction (Zhang et al. 2024). Consistent with previous studies, our results showed that Cd exposure caused tissue damage, increased MDA content, decreased GPX1 and SOD content in rat kidneys, and increased CREA and BUN content in serum. Renal tubules have been reported to be the target of Cd-induced nephrotoxicity (Zhang et al. 2024). At the same time, histopathological examinations revealed severe morphological changes, such as tubular dilation, glomerular atrophy, epithelial cell degeneration, and increased eosinophilia in the Cd administered group. PAS staining also revealed basement membrane thickening and glomerular structural deterioration. Quantitative and semiquantitative analyses confirmed significant increases in hemorrhage and inflammatory infiltration scores in the Cd group. The results indicated that Cd exposure can cause kidney injury and proximal tubular cell damage by inducing oxidative stress. In the current study, we demonstrated that Visnagin treatment normalized oxidative stress, inflammation, serum Cre and BUN levels, and morphological changes that may reflect the kidney status in Cd-induced kidney injury.

This study demonstrates the significant therapeutic effects of visnagin against Cd-induced renal toxicity. In our study, Cd exposure, consistent with the literature, led to characteristic pathological changes in renal tissue such as tubular dilation, glomerular atrophy, epithelial degeneration, and basal membrane thickening (Ou et al. 2022). However, visnagin treatment significantly reduced these histopathological disorders and significantly alleviated Cd-related glomerular structural abnormalities. A similar protective effect was reported in a study of an acute renal injury model in which the herbal combination of Rhizoma Chuanxiong and Radix Rhei was applied; this study found that the herbal treatment inhibited tubular epithelial cell apoptosis and reduced serum creatinine and BUN levels (Li et al. 2023). Furthermore, another study on renal ischemia–reperfusion (IRI) injury showed that Fimasartan pretreatment reduced tubular cell swelling and improved renal function (Abbas et al. 2022). Similarly, in a rat-induced nephrotic syndrome model, Sanqi Qushi granules were reported to alleviate glomerular podocyte damage and reduce proteinuria (Wang et al. 2023). In a diabetic nephropathy model, Carvacrol administration was found to improve glomerular and tubular damage and normalize renal function tests (Canbaz et al. 2025).

When the mechanism of apoptosis was examined, our study showed that cadmium triggered apoptosis by increasing the Bax/Bcl-2 ratio, while Visnagin supported cell survival by decreasing this ratio. This finding is consistent with the results of Rao et al., which showed that Visnagin decreased Bax expression and increased Bcl-2 expression in brain tissue (Rao et al. 2022). The apoptosis-inducing effect of cadmium has also been demonstrated in other tissues; for example, in rat hippocampus and cortex, cadmium administration was found to decrease Bcl-2 mRNA levels while increasing Bax mRNA levels and caspase-3/7 activity (Mahdavi et al. 2018). Similarly, it has been reported that in rats consuming high doses of Chaga mushrooms, Bax-positive cells increased and Bcl-2-positive cells decreased in the kidneys, and consequently, the number of TUNEL-positive apoptotic cells increased (Lee et al. 2025). In cadmium-induced testicular damage, Topiramate was shown to decrease caspase-3 activity and Bax immunostaining, while increasing Bcl-2 (Arab et al. 2022). Lithium treatment has also been reported to inhibit germ cell apoptosis in cadmium-treated testes by reducing Bax and increasing Bcl-2 (Al-Azemi et al. 2010). In kidney tissue, Carvacrol, Fimasartan, and Sanqi Qushi granules were found to inhibit apoptosis by maintaining the Bax/Bcl-2 balance, exhibiting nephroprotective properties similar to the effect of Visnagin in our study (Abbas et al. 2022; Wang et al. 2023; Canbaz et al. 2025).

The kidney is the organ responsible for the excretion of metabolic products such as creatinine, urea, and uric acid, and cadmium exposure has previously been shown to primarily affect renal function (Ayhan et al. 2025). In our study, BUN and CRE levels were examined to assess renal function. While elevated BUN is considered an indicator of serious renal lesions such as glomerulonephritis, creatinine primarily reflects glomerular filtration; it increases when glomerular filtration decreases (Liu et al. 2019). In our results, serum BUN and CRE levels were significantly increased as a result of cadmium exposure. These findings are consistent with previous studies showing that cadmium causes serious damage to the glomeruli and leads to renal dysfunction reflected by elevated biomarkers (Liu et al. 2019; Zhang et al. 2024; Ayhan et al. 2025). Treatment with visnagin in combination with cadmium chloride resulted in a significant improvement in BUN and creatinine parameters. This is an indication of visnagin's protective effect on renal function and its therapeutic role against cadmium-induced nephrotoxicity.

These observations are in line with previous research indicating that Cd is a highly toxic heavy metal for the environment and severely affects various mammalian organs, especially the kidneys, and long-term exposure can lead to irreversible damage (Sanjeev et al. 2019; Ansari et al. 2021; Yan and Allen 2021; Hernández-Cruz et al. 2022; Zhang et al. 2024). The kidney is considered one of the primary target organs of Cd accumulation and toxicity, and proximal tubule cells, in particular, are the main cause of Cd-induced kidney injury (Yan and Allen 2021; Zhang et al. 2024). Similar histopathological abnormalities have been reported in the literature following Cd exposure, including loss of the renal tubular brush border, swelling, vacuolization or separation of epithelial cells, and protein coagulation in the tubular lumen (Wan et al. 2022; Zhang et al. 2024). Additionally, findings such as glomerular abnormalities, degeneration, necrosis, disappearance of the Bowman's capsule space, tubular abnormalities, increased collagen deposition, and loss of intact basement membrane have been reported in rats exposed to Cd (Ansari et al. 2021; Ijaz et al. 2023). The increase in hemorrhage and inflammatory infiltration scores in the study is consistent with Cd-induced inflammatory responses (Kayama et al. 1995; Sanjeev et al. 2019).

In our study, Cd administration resulted in a significant increase in the levels of IL-1β and IL-6, key regulators of the pro-inflammatory response. This result is consistent with the existing literature that Cd causes tissue damage by increasing inflammatory cytokine production (Ou et al. 2022; Ayhan et al. 2025). Visnagin treatment, on the other hand, significantly reduced these cytokine levels and suppressed the inflammatory response. This finding is consistent with previous studies demonstrating the anti-inflammatory properties of visnagin; it has been previously reported that visnagin regulates inflammatory signaling pathways such as NF-κB and COX-2, reduces cytokines such as IL-1, IL-6 and TNF-α, and alleviates tissue degeneration in various organ damage models (Fu et al. 2010; Gurram et al. 2022; Rao et al. 2022; Tian et al. 2022; Sagir et al. 2023; Wan et al. 2023). Visnagin administration against cadmium-induced renal toxicity has shown a significant protective effect at both histopathological and inflammatory levels.

The mechanisms underlying cadmium kidney toxicity are generally associated with oxidative stress, inflammation, apoptosis, and mitochondrial dysfunction (Ijaz et al. 2023; Zheng et al. 2024). Numerous studies indicate that all harmful pathways converge on the production of reactive oxygen species (ROS), resulting in oxidative stress, suggesting that oxidative damage is a unifying mechanism of cadmium-induced kidney toxicity and damage (Yan and Allen 2021). Oxidative stress occurs when the balance between the antioxidant system and reactive oxygen species is disrupted. The enzymatic antioxidant system, consisting of catalase (CAT), superoxide dismutase (SOD), and glutathione peroxidase (GPx), plays a key role in scavenging ROS and limiting lipid peroxidation. SOD reduces the damage caused by superoxide radicals by converting them to H₂O₂, while CAT and GSH-Px maintain cellular protection by degrading the resulting H₂O₂ into water (Zhang et al. 2023). Cadmium causes oxidative stress by increasing lipid peroxidation products such as ROS and MDA, while depleting antioxidant enzymes such as SOD, CAT, and GPx and decreasing glutathione (GSH) levels (Aqeel et al. 2020; Abdelnaby et al. 2022; Sagir et al. 2023; Zhang et al. 2024). Cadmium exposure causes significant decreases in the reduced form of GSH and CAT activity, increasing susceptibility to ROS damage (Abdelnaby et al. 2022). In addition, Cd disrupts the oxidant/antioxidant balance at the cellular level by increasing the production of nitric oxide (NO) and myeloperoxidase (MPO), which are markers of lipid peroxidation (Aqeel et al. 2020). Significant increases in lipid hydroperoxide (LOOH), protein carbonyl content (PCC), total oxidant status (TOS), and oxidative stress index (OSI) levels were observed in Cd-poisoned rats (Sanjeev et al. 2019). Indeed, numerous studies in the literature have shown that Cd increases oxidant compounds while decreasing antioxidant capacity (El-Sharaky et al. 2007; Su et al. 2021). Similarly, the significant decrease in SOD and GPX1 levels observed in the Cd group in this study supports the antioxidant defense-suppressing effect of cadmium, and the re-elevation of both enzymes in the Vis + Cd group demonstrates visnagin's protective role on oxidative stress. The antioxidant capacity implied by the protective effects of visnagin is consistent with studies on other natural compounds that ameliorate Cd toxicity by neutralizing oxidative stress (Sanjeev et al. 2019; Aqeel et al. 2020; Ansari et al. 2021). Furthermore, the significantly increased MDA levels in the Cd group were significantly reduced by vis administration, suggesting that visnagin attenuates oxidative damage by suppressing lipid peroxidation.

The decrease in inflammatory infiltration scores observed in our study suggests that Visnagin limits tissue damage by modulating the inflammatory response. Cd exposure is known to trigger a proinflammatory response, leading to the accumulation of inflammatory cells in the tissue; this is revealed histologically by increased inflammatory infiltration scores. The decrease in scores with Visnagin administration supports the control of the Cd-induced inflammatory process and the significant anti-inflammatory effect of Visnagin (Gurram et al. 2022; Rao et al. 2022). Cd is known in the literature to trigger both apoptosis and mitochondrial dysfunction, leading to disruption of the mitochondrial electron transport chain and increased ROS production (Yan and Allen 2021; Sagir et al. 2023). Kidneys are particularly sensitive to Cd toxicity because they require a large number of mitochondria, and Cd has been reported to disrupt mitochondrial ultrastructure (Hernández-Cruz et al. 2022; Ijaz et al. 2023). The protective effect of Visnagin may contribute to reducing tissue damage by alleviating this mitochondrial dysfunction.

**Fig. 6:**
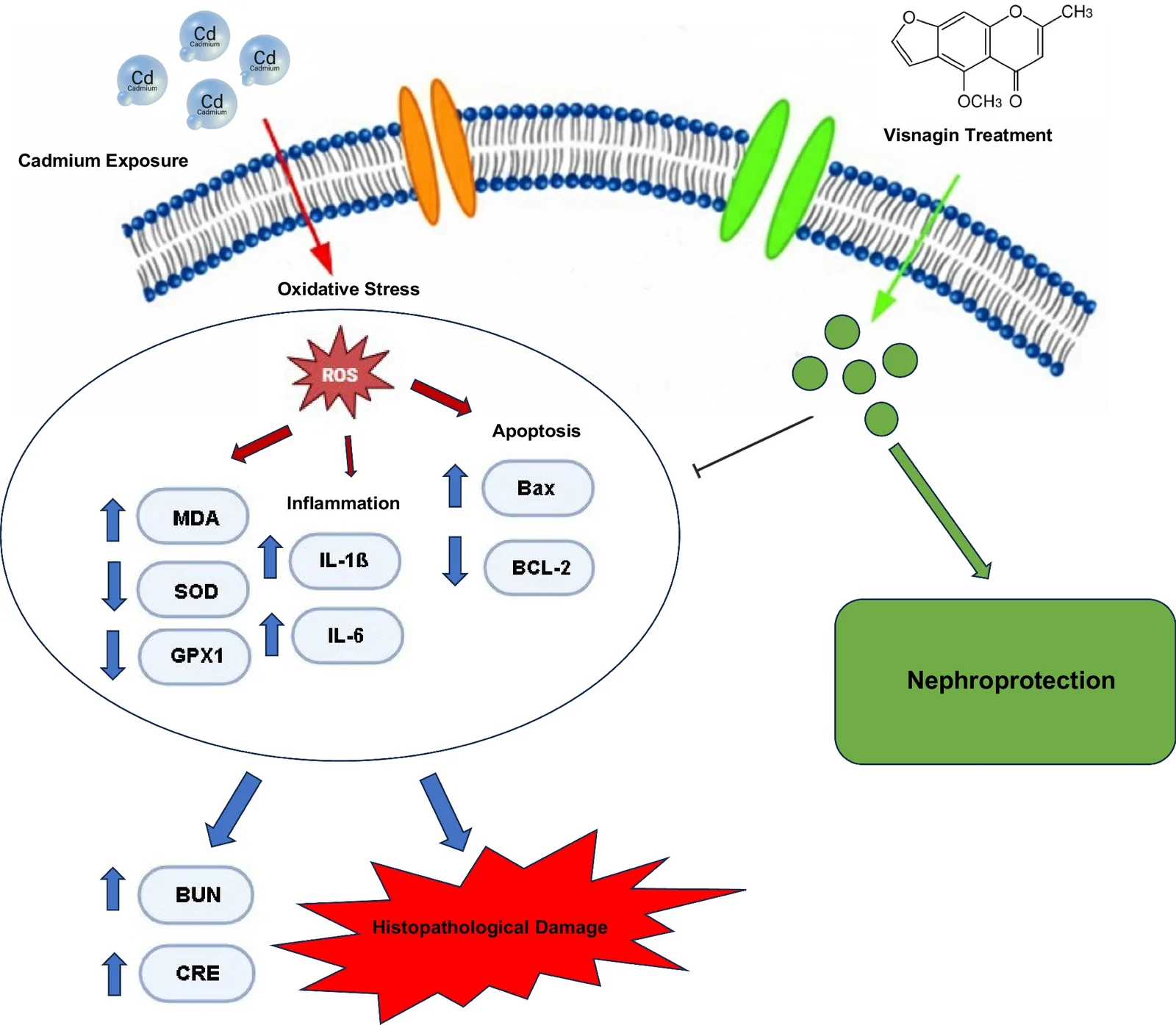
Proposed mechanistic scheme of visnagin’s nephroprotective effect against cadmium toxicity

Our study shows that cadmium exposure causes both histological and functional abnormalities in kidney tissue. Cadmium exposure caused significant histological damage in the kidney tissue, including tubular dilatation, glomerular atrophy, epithelial degeneration, and basement membrane thickening. In addition, the cadmium group showed both structural and functional toxic effects with increases in bleeding and inflammatory infiltration scores, elevations in renal function markers BUN and CRE, increases in MDA, and decreases in SOD and GPX1 activities. Visnagin treatment significantly attenuated these histological changes and limited tissue damage by reducing inflammatory infiltration and bleeding scores. Biochemically, Visnagin attenuated oxidative stress by decreasing MDA levels and increasing SOD and GPX1 activities, and supported the preservation of renal function by normalizing the increases in BUN and CRE. In the Visnagin-only group, similar normal histological structures were observed as in the control group, and no inflammatory or hemorrhagic changes were observed; This suggests that Visnagin is safe at recommended doses and does not cause adverse effects on the kidneys. All these findings suggest that Visnagin has a protective effect against Cd toxicity through histological, oxidative, and inflammatory mechanisms and supports the structural and functional integrity of renal tissue (Fig. 6). Future research may clarify its nephroprotective potential and clinical applicability by further examining the biochemical and molecular pathways that Visnagin modulates in Cd-induced kidney injury, its effects on oxidative stress markers, and specific apoptotic or inflammatory pathways.

## Conclusion

Our study demonstrates that cadmium exposure causes both structural and functional abnormalities in kidney tissue. Histologically, tubular dilation, glomerular atrophy, and epithelial degeneration were observed; increased inflammatory infiltration and hemorrhage, along with elevated BUN and creatinine levels, and impaired antioxidant defenses (decreased SOD and GPX1, increased MDA) were observed. In addition, Visnagin treatment reversed the cadmium-induced imbalance between pro-apoptotic Bax and anti-apoptotic Bcl-2 proteins, thereby promoting cellular survival in renal tissues. Visnagin treatment significantly attenuated these changes, limiting tissue damage, reducing oxidative stress, and preserving renal function. These findings suggest that Visnagin has a multifaceted protective effect against Cd toxicity and may be a potential nephroprotective agent in maintaining renal health.

### Limitations

While this study presents some evidence regarding the nephroprotective potential of Visnagin, certain limitations must be considered. First, our findings are based on a rat model with specific dose and duration parameters; therefore, caution should be exercised in generalizing these results to human clinical settings or different exposure scenarios. Additionally, although we focused on key apoptotic and oxidative markers, the broader molecular picture, including specific inflammatory cytokines and detailed epigenetic modulations, has not yet been fully mapped. Further research involving a wider dose range, longer exposure times, and advanced molecular techniques (such as proteomics or gene silencing) is important to comprehensively elucidate the mechanism of action of Visnagin and validate its safety as a therapeutic agent in clinical nephrology.

## Data Availability

All source data for this work (or generated in this study) are available upon reasonable request.
